# Attitudes towards tooth fillings in Tanzanian adults and its association with previous filling experience

**DOI:** 10.1186/s12903-018-0474-x

**Published:** 2018-01-18

**Authors:** Kasusu K. Nyamuryekung’e, Satu M. Lahti, Risto J. Tuominen

**Affiliations:** 1 School of Dentistry, Department of Orthodontics, Paedodontics and Community Dentistry, Muhimbili University of Health and Allied Sciences, Dar es Salaam, Tanzania; 2 Department of Community Dentistry, University of Turku, Turku, Finland; 3 Turku Clinical Research Centre, Turku University Hospital, Turku, Finland; 4 Hospital District of Southwest Finland, Turku University Hospital, Turku, Finland; 5 Department of Public Health, University of Turku, Turku, Finland; 6 School of Dentistry, University of Namibia, Windhoek, Namibia

**Keywords:** Attitudes, Restorative services, Utilization

## Abstract

**Background:**

Tooth filling treatment is utilized at low levels in many low and middle-income countries (LMICs), further, little is known about the prevailing attitudes towards such treatment. This study aimed to assess attitudes towards tooth filling among Tanzanian adults and how previous tooth filling experience is associated with these attitudes.

**Methods:**

A pretested structured questionnaire was distributed among 1522 out-patients in four regional hospitals in Tanzania in 2015–16. The questionnaire had eight statements on a 6-point Likert scale measuring attitudes towards tooth filling. Responses were analyzed independently and through a constructed attitude sum score. Linear regression analysis was used to assess the association of previous tooth fillings on attitudes towards tooth filling treatment.

**Results:**

The respondents were mostly female (57.3%), with a mean age of 33.1 years (SD 11.3). About one third of the respondents (36.4%) had primary level of education. Attitudes towards tooth filling treatment were generally negative. Low levels of education and income were associated with more negative attitudes. A small proportion (11.5%) had a previous tooth filling. Having a previous tooth filling was associated with a more positive attitude towards tooth fillings regardless of socioeconomic status.

**Conclusions:**

This study shows that even in areas with limited resources and availability of services, previous experience of tooth fillings is related to more positive attitudes towards restorative treatment, which should be taken into account when planning oral health care programs.

**Electronic supplementary material:**

The online version of this article (10.1186/s12903-018-0474-x) contains supplementary material, which is available to authorized users.

## Background

A significant proportion of populations in low and middle-income countries (LMICs) suffer from oral diseases, leading to poor oral health status [1]. However, preventive dental services are rarely utilized in these settings [2]. As a result, patients frequently present at the health facilities due to symptomatic reasons [3, 4]. Toothache due to dental caries is the most common reason for attendance in dental facilities in LMICs [3, 5, 6] and the most common dental treatment rendered has been tooth extractions and not fillings [1, 7–9]. This often leads to premature loss of teeth and poor quality of life [10]. An increase in rates of utilization of tooth filling services in LMICs may result into an improvement in oral health status and health generally.

Several theories have attempted to describe health behaviours and factors influencing them [11, 12]. Even though they differ greatly in their scope and applicability, most are in accord with existence of both internal and external factors that determine attitudes and behaviours. One of the well-known models describing health care utilization is by Andersen et al. [13]. This model looks at dimensions (predisposing, enabling and need factors) behind access to and utilization of health services once an individual becomes ill. Predisposing factors are those present prior to onset of illness and describe the predilection of individuals to use health services. The enabling factors describe the individual’s (financial) means available for use on health services whereas need for care refers to (subjective) level of experienced illness [13].

Predisposing factors of individuals determine their beliefs regarding seeking of healthcare services and types of health care utilized. The ensuing tendency for favourable or unfavourable response to a health need is defined as “attitude” [14]. Patients’ beliefs towards a particular health service may influence their utilization. This belief can be considered as a reflection of a person’s perceptions, feelings and knowledge regarding available health care services. Such beliefs and misinformation, and lack of knowledge on restorative care have been identified as some of the key obstacles towards utilization of tooth filling services in LMICs [15].

It has been suggested that attitudes in utilization of any oral health service are characterized by subjective valuation in terms of convenience, appropriateness, cost and the perceived quality of the care which will be received [13]. Furthermore, these attitudes are not considered to be static, but change depending on variations in both internal and external factors [16]. Factors such as perceived values of oral health care, levels of oral health knowledge and attitudes, educational status and income have been shown to influence utilization of oral health services [17].

Despite attempts to introduce restorative services within the oral health systems in low income countries, the success of these endeavours has been limited [18, 19]. Possible barriers to utilization of these services by patients include lack of knowledge of restorative care, unavailability of materials and equipment, cost of services and negative past experiences with dental treatment [3, 19, 20]. Nevertheless, acceptability and utilization of tooth filling services is partly dependent on the prevailing attitudes of patients towards the service [17, 21–23]. If the patients have negative attitudes towards tooth filling services, the rate of its utilization is likely to also be low, irrespective of service availability.

The aim of this study was to determine the attitudes of Tanzanian patients towards tooth filling treatment and how previous fillings are associated with them.

## Methods

### Setting

This study was conducted in four government regional hospitals in Tanzania. These hospitals are categorized as the top referral hospitals within their respective regions by the Tanzanian Ministry of Health [24]. To represent typical regional hospitals, Amana, Vwawa, Sekou-toure and Mawenzi hospitals in Dar es salaam, Mbeya, Mwanza and Kilimanjaro regions respectively, were selected.

### Sampling

Because previous information on the distributions of patients’ attitudes was not available, quota sampling was employed. The enrolment period at each hospital was one month. A total of 400 outpatients from each hospital were considered sufficient for study purposes.

### Development of the questionnaire

No study tool was available which could be used to measure attitudes towards tooth filling treatment in this setting. Therefore, a questionnaire was developed in the local language, Kiswahili. Questionnaire development was based on guidelines by Norman and Streiner, 2008 [25].

In the first phase, an exploratory pilot study to assess the prevailing beliefs and feelings towards tooth filling services amongst dental outpatients was conducted. The study consisted of open ended questions which aimed to determine attitudes regarding tooth fillings. It was carried out amongst 79 dental patients attending a university dental clinic, not included in the main study. Of the responses statements, 34 directly related to beliefs on tooth-filling treatment were identified.

In the second phase, the identified statements were edited and rephrased; by transforming them from their specific and personal point of view into broad, widely-applicable statements. Thereafter, a 6-point Likert scale was included for each statement: (1) completely agree to (6) completely disagree, and compiled into a questionnaire. This was administered among 58 different dental patients and their responses to the statements were analyzed as a scale. The scale was tested for variability in responses, item total correlations and internal consistency. Statements with high endorsement rates (more than 80% of the respondents selecting the same response alternatives) and with missing values exceeding 20% were omitted. To ensure homogeneity of the statements; those with negative correlation to the scale and with Pearson *r’*s score of less than 0.20 were also omitted. Internal reliability of the scale (Cronbach’s alpha) was 0.799.

The final questionnaire had a total of 8 attitude statements (Table 1) remaining from the initial 34. Additional questions on previous tooth filling history and socio-demographic variables; sex, age, level of education, marital status, monthly household income and payment modality used for health services were included (Additional file 1).

**Table 1:** Overall and statement- specific mean scores and confidence intervals (95 CI) of statements measuring attitude towards tooth fillings

Attitude statements	n	Mean score	95 CI
Tooth extractions are the only effective means of treating a toothache	1472	−1.23	− 1.33, − 1.12
Despite tooth fillings, you will have to extract your tooth eventually	1461	− 1.07	− 1.17, −.97
Tooth fillings cause harm by being the source of another toothache	1462	−0.99	− 1.09, −.90
Tooth fillings cause filled tooth to fracture	1456	−0.12	−.22, −.01
Tooth fillings are expected to dislodge after a short while	1453	−0.06	−.17, .04
Tooth fillings are better than extractions for management of toothaches	1478	0.03	−.08, .92
Tooth fillings completely eliminate toothache	1468	0.62	.52, .71
Tooth fillings allow an individual to eat hot/cold foodstuffs with no problem	1453	0.83	.73, .92
Overall attitude scores	1389	−1.99	−2.42, −1.55

### Data items

Responses for positively worded attitude statements scored from + 3 (completely agree) to − 3 (completely disagree). Negatively worded statements were reverse-coded before analysis. Therefore, each statement scored from + 3: highly positive attitude to − 3: highly negative attitude. The sum of these scores from 8 statements served as the final attitude-sum score for each respondent. Hence, the scale had a possible range of scores from − 24 to + 24.

Determination of previous tooth-filling experience was by asking “*How many teeth have you had filled*?” The responses were dichotomized as: 0) never had a filling and 1) had at least one filling.

Age of the respondents ranged from 18 to 82, but was categorized into four groups; 1) 18–24, 2) 25–34, 3) 35–44 and 4) 45 and above. The respondents’ highest level of education was categorized into three levels as 0) Low (no formal education, primary school), 1) Intermediate (did not finish secondary school, finished secondary school) and 2) High (college/university). Monthly household income in Tanzanian shillings (Tshs) comprised six levels but were categorized into three; 0) 100,000 or less, 1) 110,000–500,000 and 2) 550,000 and above; *1US$ = 2187 Tshs, August 2016*. Patients’ health insurance status was determined and categorized into 0) out of pocket and 1) health insurance.

### Data collection

Patients were approached by research assistants at the waiting area, informed of the purpose of the study and invited to participate. Research assistants provided instructions and clarifications on their filling and responded to any questions. Data was collected by means of a self-administered questionnaire in the hospitals’ waiting rooms.

### Data analysis

Statistical differences in proportions were compared using chi-square tests; independent samples t-test and analysis of variances were used to test differences between the mean values.

Linear regression analysis was used to explore the associations of socio-demographic variables and previous tooth fillings with attitudes on tooth filling treatment. The assumptions for linear regression were observed. In Block 1: sex, age, education, income and health insurance were added. In Block 2: having a previous tooth filling was added. All analyses were conducted using SPSS for Windows, Version 20; statistical significance was set at *p* < 0.05.

### Ethical considerations

Approval for this study was obtained from Ethical Committee of the Muhimbili University of Health and Allied Sciences (2015–06-12/AEC/Vol. IX/108). Ethical permission to conduct this study in regional hospitals was obtained from the regional administrative secretaries of the respective regions (Dar es Salaam, Mwanza, Kilimanjaro and Mbeya). Permission was also obtained from the medical officers’ in-charge of the regional hospitals. Participants were given verbal and written information about the study and confidentiality was assured. Signed, informed consent was obtained from all participants.

## Results

A total of 1522 respondents from four regional hospitals participated in this study. The respondents were mostly female (57.3%), with a mean age of 33.1 years (SD 11.3). About one third of the respondents (36.4%) had primary level of education. Overall mean attitude scores towards tooth filling treatment were negative (Table 1). Majority of the respondents perceived that tooth extractions were the only effective means of treating a toothache and that despite tooth fillings, a tooth will have to be extracted eventually. On the other hand, the views that tooth fillings completely eliminate toothache and allow an individual to eat hot/cold foodstuffs with no problem were perceived most positively.

Respondents with low levels of education and monthly income had more negative attitudes towards tooth filling treatment. On the contrary, those with previous tooth filling experience had more positive attitudes towards the service compared to others (Table 2). Women, users of health insurance, and those with high levels of education and income had correspondingly higher proportions reporting to have ever had a tooth filling (Table 3).

**Table 2:** Distribution of respondents and their variation in attitudes towards tooth filling treatment

	n (%)	Mean attitude scores	95 CI	*P*-value
Sex				
Male	645 (42.7)	−1.96	−2.66, − 1.25	.847
Female	866 (57.3)	−2.04	−2.60, −1.49	
Age				
18–24	373 (25.0)	−1.08	−1.98, −.18	.020
25–34	522 (35.0)	−2.86	−3.58, − 2.14	
35–44	368 (24.7)	− 1.88	−2.77, − 1.00	
45 and above	229 (15.3)	− 1.58	−2.79, −.38	
Marital status				
Single	546 (36.6)	−2.02	−2.76, −1.28	.063
Married	769 (51.6)	−1.61	−2.22, −.99	
Others	175 (11.7)	−3.31	− 4.55, −2.08	
Education				
Low	548 (36.4)	−3.20	−3.95, −2.45	.000
Intermediate	642 (42.6)	−1.52	−2.17, −.86	
High	316 (21.0)	−0.87	−1.81, −.07	
Monthly household income (Tshs)				
Low (≤ 100,000)	593 (40.3)	−2.53	−3.28, −1.79	.019
Mid (110,000–500,000)	654 (44.4)	−1.96	−2.60, − 1.33	
High (≥ 510,000)	226 (15.3)	−0.64	−1.70, .41	
Payment modality				
Out of Pocket	1101 (74.2)	−2.22	−2.73, −1.70	.078
Health insurance	382 (25.8)	−1.31	−2.18, −0.45	
Previous tooth filling				
Yes	162 (11.5)	1.00	−.50, 2.50	.000
No	1214 (88.5)	−2.37	−2.87, −1.92

**Table 3:** Variation in previous tooth filling history by respondents’ background factors

	Have you ever had a tooth filling?		*p* value
	Yes *n (%)*	No *n (%)*	
Sex			
Male	58 (9.2)	573 (90.8)	.021
Female	113 (13.2)	743 (86.8)	
Age			
18–24	34 (11.6)	329 (88.4)	.094
25–34	60 (11.7)	453 (88.3)	
35–44	31 (8.6)	331 (91.4)	
45 and above	34 (15.4)	187 (84.6)	
Marital status			
Single	76 (14.1)	462 (85.9)	.068
Married	76 (10.0)	682 (90.0)	
Others	18 (10.5)	153 (89.5)	
Education			
Low	26 (4.9)	508 (95.1)	.000
Intermediate	64 (10.1)	570 (89.9)	
High	78 (24.8)	236 (75.2)	
Monthly household income (Tshs)			
Low (≤ 100,000)	46 (7.9)	538 (92.1)	.000
Mid (110,000–500,000)	71 (10.9)	578 (89.1)	
High (≥ 510,000)	44 (19.9)	177 (80.1)	
Payment modality			
Out of Pocket	86 (7.9)	1003 (92.1)	.000
Health insurance	83 (22.3)	289 (77.7)	

The only background factor significantly associated with positive attitudes towards tooth filling was the level of education. Nevertheless, even after controlling these factors, having a previous tooth filling remained positively associated with attitudes towards tooth filling services (Table 4). However, the chosen independent variables could explain only a small proportion of the total variance in attitudes.

**Table 4:** The association between previous tooth filling and attitudes towards tooth filling treatment

Variables	Block 1 (β)	Block 2 (β)
Sex (Male/Female)	.005 (−.80, .99)	−0.04 (−.97, .82)
Age	.016 (−.03, 0.52)	.007 (−.04, .05)
Education (Low/Medium/High)	.099 (.40, 1.81)**	.076 (.14, 1.56)*
Income (Low/Intermediate/High)	.032 (−.33, 1.08)	.032 (−.32, 1.08)
Payment modality (Out of Pocket/ Health insurance)	.003 (−1.09, 1.16)	.011 (−1.31, .885)
Have you ever had a tooth filling (Yes/No)		.113 (1.52, 4.37)**
F	3.616**	5.763**
R^2^	.013	.025
ΔR^2^	.013**	.012**

* p < 0.05

** p < 0.01

## Discussion

Negative attitude towards dental care has been shown to result in deteriorations in oral health, regardless of socio-demographic and educational status [22]. Current study reveals that most of the respondents had negative attitudes towards tooth filling services. This may indicate both an overall dissatisfaction and uncertainty regarding successful outcomes of treatment or a general lack of awareness of tooth filling services. Commonly, these filling services in LMICs are rendered in a health care environment experiencing recurrent absence of dental materials, equipment and manned by and insufficient workforce that is not wholly confident in providing such services [9, 18, 24]. Therefore, the available oral health services are mostly focused on pain relief, emergency care and tooth extractions [2]. As a result, significant proportions of the population might end up viewing tooth extraction services as the first line and the desirable modality of treatment for toothaches due to high frequency of their provision by dental care providers. Accordingly, this may undermine their appreciation of tooth filling services because most have only ever been exposed to tooth extractions.

Education, income and health outcomes have been demonstrated to be positively correlated through numerous studies [26–29]. Current findings indicate that those with high levels of education and income had more positive attitudes and concurrently higher proportions with previous tooth fillings. It has been suggested that those who have a high level of education have an increased likelihood of acquiring and assimilating new information, and to seek and obtain correct knowledge on health services [30]. It has also been suggested that knowledge may be an important constituent during attitude formation [31], and its provision influences dental attitudes [32]. Therefore, observed positive attitudes in these sub-groups could be a reflection of their capability of obtaining correct information, and subsequent ability to afford tooth filling services when required.

Several studies have identified cost of services as one of the major barriers towards utilization of tooth filling services in Tanzania [15, 19]. As a result of lack of universal health insurance scheme, most patients currently pay for their health care through out of pocket payments in Tanzania [33]. Availability of health insurance subsidizes costs incurred by patients and encourages seeking and utilization of appropriate oral health care services [26, 34]. Considering that restorative services generally cost more than tooth extractions in this setting [9], affordability is likely to play a significant role in determining utilization of tooth filling services. Several studies have demonstrated how affordability of tooth filling services influence their utilization [17, 21, 35, 36]; and its role is likely to be even larger in a resource constrained environment such as this one. This may explain current findings showing that patients with health insurance and high levels of income had higher proportions utilizing tooth filling services compared to those paying directly out of pocket.

The model used in the current study observed low levels of association between previous tooth fillings and attitudes towards tooth filling services. It seems that there are more important and stronger factors which were not captured in the current model. Therefore, our model is likely to be only a partial representation of the complex reality. More research and comprehensive models are needed before the magnitude of effects of each background factor can be determined. Nevertheless, in this study population, previous tooth fillings were shown to be positively associated with positive attitudes towards tooth filling services.

Overall, only a small proportion of Tanzanians have ever utilized tooth filling services [3, 37, 38], as corroborated by our findings. This is similar to observations from many other LMICs [3, 7, 9, 39]. The finding that those reporting to have had previous tooth fillings were associated with a comparatively more positive attitude has two possible interpretations. Firstly, patients with a good attitude towards the services utilized it more frequently. This is substantiated by findings that favourable attitudes towards dental care have been demonstrated to be associated with more frequent preventive and restorative visits [22]. However, the decision to utilize tooth filling services is a complex one that does not rely solely on patient attitudes. Costs of service, availability of services and previous experiences have been shown to influence usage of these services [15, 20]. Other factors include dentist’s preferences and expertise, the prognosis of the tooth and availability of resources amongst many others [9, 40].

Secondly, patients that received tooth filling services developed resultant positive attitudes. This may indicate patient satisfaction and approval of provided services. Indeed, there have been recent reports of high satisfaction levels amongst patients who received tooth filling services in this setting [41]. However, due to the current study design it was not possible to ascertain whether subjects had positive attitudes towards tooth filling prior to undergoing tooth filling treatment, or that the observed positive attitudes are a result of the treatment that they had previously received.

Findings from this study have two possible inter-linked policy implications. First, provision of more tooth fillings in a resource constrained setting can be a promoting factor for attitude favoring having tooth fillings in future. Second, individuals who have undergone tooth filling within a resource constrained setting may develop favorable attitudes towards the service. There have been previous efforts in increasing the availability of tooth filling services in Tanzania with limited success [19]. However, the provision of this service still remains far below the expected levels [18, 37]. Modification of the environment by removal of external barriers may function to encourage attitude and accompanying behavioral change [42]. Therefore, ensuring a constant and reliable availability of tooth filling materials and equipment is important [9]. This may stimulate regular provision of this service to the patients and serve as a practical emphasis of its role as the first line of treatment for toothaches.

Interpretations of findings from this study have to be made with the following limitations in mind; Firstly, there was no instrument for measuring attitudes towards tooth filling treatment for African societies and health care settings, therefore, we had to develop one. The questionnaire development process allowed researchers to analyze responses elicited from open-ended questions and utilize them in formulation of draft questionnaires. Consequently, the statements included within the scale are solely derived from participants that responded to the open-ended questions. Although care was taken to ensure that data was collected until the point of saturation, it is possible that a different set of statements would be elicited if a different sample of participants had given their responses. In addition, a statement on effectiveness of tooth extractions in management of a toothache was included onto our attitude scale. This was warranted by preliminary analyses of open-ended responses revealing that beliefs on tooth extractions as the only way of managing toothaches greatly influenced beliefs regarding tooth filling treatment. Nevertheless, taking into consideration the internal consistency of the resultant attitude scale and face validation, the instrument was considered to be sufficient for use in the study. However, it is important that other instruments are developed in other LMICs and their findings be compared with ours. It may be that some new items may arise from them and some items included in this instrument become reinforced and corroborated by other studies.

Secondly, this study was conducted amongst patients attending regional hospitals in Tanzania. These hospitals are located in urban centres of the regions. Furthermore, due to their status as referral hospitals, they charge higher registration and service fees as compared to other government hospitals within the regions. Therefore, it is probable that patients with very low incomes and from rural areas were underrepresented in the study sample. However, selection of regional hospitals was necessary as they were more likely to have functional dental units. Moreover, their large patient flow offered the greatest possibility of encountering those that know of, and have had previous tooth fillings.

## Conclusion

Utilization rates for tooth filling services are likely to remain low if the general population continues having negative attitudes towards tooth fillings. Information to general population is required in order to foster their utilization of restorative services. Community experiments are needed to study how the positive attitudes of those with previous tooth filling experience can be used to direct general population’s views more positively.

## Additional file

Additional file 1: Informed consent form and questionnaire. Informed consent form and the accompanying questionnaire used to collect data for the manuscript “Attitudes towards tooth fillings in Tanzanian adults, and how previous experience in filling services is associated with them”. (DOCX 31 kb)

## Abbreviations

LMICs: Low and middle-income countries
SD: Standard deviation
95 CI 95%: confidence intervals
