# Supplementary material for: Attitudes towards tooth fillings in Tanzanian adults and its association with previous filling experience
Source: BMC Oral Health. 2018 Jan 18;18:12. doi: 10.1186/s12903-018-0474-x (PMC5774145; doi:10.1186/s12903-018-0474-x)
Supplement: Additional file 1: — Informed consent form and questionnaire. Informed consent form and the accompanying questionnaire used to collect data for the manuscript “Attitudes towards tooth fillings in Tanzanian adults, and how previous experience in filling services is associated with them”. (DOCX 31 kb) [file 12903_2018_474_MOESM1_ESM.docx]

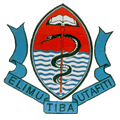
**MUHIMBILI UNIVERSITY OF HEALTH AND ALLIED SCIENCES**

**DIRECTORATE OF RESEARCH AND PUBLICATIONS**

**INFORMED CONSENT FORM**

**ID-NO: _________________**

**Consent to participate in a research.**

Greetings! You have been selected to participate in a research project with the objective of determining patient factors influencing utilization and their perception on outcomes of tooth filling services in government health facilities.

**What participation involves**

If you agree to participate in the study, you will be asked to respond to statements on your overall previous dental experience, your understanding of dental filling services and an assessment of your willingness to pay for dental services.

**Confidentiality**

All information we collect on forms will be entered into computers with only study identification number. Any information given will in no way influence your treatment or the payment of your treatment.

**Risks**

We do not expect that any harm or injury will happen to you because of joining this study.

**Rights to withdraw**

Taking part in this study is entirely your choice. You can stop participating in this study at any time, even if you already have given consent. Refusal to participate or withdraw from the study will not involve penalty or any loss on your part.

**Benefits**

If you agree to take part in this study, you will be offering information that is valuable towards betterment of dental services in Tanzania. As an important stakeholder within the health sector, the information you offer will provide important insights into the oral health service delivery patterns, as well as allow areas of intervention for improvement

**Who to Contact**

If you ever have questions about this study, you should contact the principal investigator, Dr. Kasusu Klint Nyamuryekung’e, Muhimbili University of Health and Allied Sciences, (P.O Box 65013, Dar es Salaam). If you ever have questions about your rights as a participant, you may call Prof. Mainen J. Moshi, Chairman of the Senate Research and Publications Committee, P.O. Box 65001, Dar es Salaam. Tel: 2150302-6, 2152489.

**Signature**

Signature of participant: ________________

Hospital name: ________________________

Date: _______________________________

**SECTION I: Please fill in this section by filling-in or circling the applicable alternative**

1. Age (years): _____________

2. Sex:

1. Male
2. Female

3. Marital status:

1. Single
2. Co-habiting
3. Married
4. Widowed

4. Highest education level attained:

1. Never attended school
2. Primary school
3. Did not complete secondary school
4. Completed secondary school
5. College/University

5. How many people are currently living in your household? ______________________

6. The average monthly income of your household best fits within which category?

1. Below Tshs 100,000
2. Tshs 110,000 to Tshs 250,000
3. Tshs 260,000 to Tshs 500,000
4. Tshs 510,000 to Tshs 750,000
5. Tshs760,000 to Tshs 1,000,000
6. Above Tshs 1,000,000

7. Is a dental problem the reason for your visit to the hospital today?

1. Yes
2. No

8. What is your modality for payment of health services today?

1. Out of pocket
2. NHIF
3. NSSF
4. CHF
5. Exempted
6. Others: ________________________

9. How would you describe the state of your teeth?

1. Very poor
2. Poor
3. Good
4. Very good

10. How satisfied are you with the appearance of your teeth?

1. Very satisfied
2. Satisfied
3. Unsatisfied
4. Very unsatisfied

11. Have you ever gone to a hospital because of a toothache?

1. Yes
2. No

12. Have you ever had a toothache?

1. Yes
2. No

13. Does this facility offer the following services?

| 1) Tooth extraction | 1). Yes | 2). No | 3). Don’t know |
| --- | --- | --- | --- |
| 2) Tooth filling | 1). Yes | 2). No | 3). Don’t know |
| 3) Scaling and root planing | 1). Yes | 2). No | 3). Don’t know |
| 4) Denture fabrication | 1). Yes | 2). No | 3). Don’t know |

14. Have you ever had an extraction of one of the following teeth?

| 1) Upper anterior tooth | 1). Yes | 2). No |
| --- | --- | --- |
| 2) Upper posterior tooth | 1). Yes | 2). No |
| 3) Lower anterior tooth | 1). Yes | 2). No |
| 4) Lower posterior tooth | 1). Yes | 2). No |

*When responding to questions* ***15*** *and* ***16****, please run your tongue across your teeth in the oral cavity to assist you in counting the number of teeth present in each jaw.*

15. How many teeth do you have in your upper jaw? _____________

16. How many teeth do you have in your lower jaw? _____________

17. Have you ever had a filling on one of the following teeth?

| 1) Upper anterior tooth | 1). Yes | 2). No |
| --- | --- | --- |
| 2) Upper posterior tooth | 1). Yes | 2). No |
| 3) Lower anterior tooth | 1). Yes | 2). No |
| 4) Lower posterior tooth | 1). Yes | 2). No |

18. How many teeth have you ever had filled so far? __________________ (If none, write “0”)

**SECTION II: Read each statement carefully and circle the alternative which is applicable to *you.* Please note, this is not an examination; there is no single correct answer.**

1. Tooth filling treatment completely eliminates toothache
2. Completely agree
3. Agree
4. Mildly agree
5. Mildly disagree
6. Disagree
7. Completely disagree
8. Tooth fillings are expected to dislodge after a short while
9. Completely agree
10. Agree
11. Mildly agree
12. Mildly disagree
13. Disagree
14. Completely disagree
15. Tooth fillings may cause harm later by causing another toothache
16. Completely agree
17. Agree
18. Mildly agree
19. Mildly disagree
20. Disagree
21. Completely disagree
22. Tooth fillings lead to a subsequent slow fracture of filled teeth
23. Completely agree
24. Agree
25. Mildly agree
26. Mildly disagree
27. Disagree
28. Completely disagree
29. A filled tooth allows one to eat cold/hot foodstuffs without any problems
30. Completely agree
31. Agree
32. Mildly agree
33. Mildly disagree
34. Disagree
35. Completely disagree
36. Despite receiving tooth fillings, you will have to extract the tooth eventually
37. Completely agree
38. Agree
39. Mildly agree
40. Mildly disagree
41. Disagree
42. Completely disagree
43. Extraction is the only sure way to treat toothache
44. Completely agree
45. Agree
46. Mildly agree
47. Mildly disagree
48. Disagree
49. Completely disagree
50. Tooth fillings are better treatment for toothaches than tooth extractions
51. Completely agree
52. Agree
53. Mildly agree
54. Mildly disagree
55. Disagree
56. Completely disagree

****Thank you very much for your participation****
